# Arbutin protects against methotrexate-induced pulmonary injury in rats via modulation of oxidative stress, inflammation, and ER stress

**DOI:** 10.3389/fvets.2025.1680886

**Published:** 2025-10-30

**Authors:** Nihal Turkmen Alemdar, Selim Demir, Esin Yulug, Nadire Sevdenur Erdogan, Elif Ayazoglu Demir, Ahmet Mentese, Yuksel Aliyazicioglu

**Affiliations:** ^1^Department of Medical Services and Techniques, Vocational School of Health Services, Recep Tayyip Erdogan University, Rize, Türkiye; ^2^Department of Medical Biochemistry, Faculty of Medicine, Karadeniz Technical University, Trabzon, Türkiye; ^3^Department of Histology and Embryology, Faculty of Medicine, Karadeniz Technical University, Trabzon, Türkiye; ^4^Department of Chemistry and Chemical Processing Technologies, Macka Vocational School, Karadeniz Technical University, Trabzon, Türkiye

**Keywords:** arbutin, endoplasmic reticulum stress, methotrexate, Nrf2, pulmonary toxicity, SIRT1

## Abstract

**Introduction:**

Methotrexate (MTX) is a widely utilized agent in the treatment of cancer, yet it is notable that it can induce pulmonary toxicity in cases of high-dose chemotherapy. Arbutin (ARB) is a hydroquinone compound that is present in members of the Lamiaceae, Ericaceae and Rosaceae families, and experimental studies have demonstrated its capacity for lung protection. The present study aimed to determine whether ARB could reduce the pulmonary toxicity of MTX and to explore the underlying mechanisms.

**Methods:**

The lung toxicity rat model was created by means of a single intraperitoneal injection of MTX at a dose of 20 mg/kg. The animals were then treated with two different doses of ARB (50 and 100 mg/kg) for a period of 7 days. Following the conclusion of the treatment period, a histopathological examination of the lung tissue samples was conducted. The remaining tissue samples were evaluated for oxidative stress (OS), inflammation, endoplasmic reticulum stress (ERS), sirtuin 1 (SIRT1)/nuclear factor erythroid-related factor 2 (Nrf2) pathway, and apoptosis for further analysis.

**Results:**

The administration of MTX resulted in the inhibition of SIRT1/Nrf2 in lung tissue, accompanied by an escalation in OS, inflammation, ERS, and apoptosis levels. This was concomitant with a significant enhancement in the severity of histopathological findings. Nevertheless, ARB reversed MTX-induced biochemical and pathological changes through SIRT1/Nrf2 modulation.

**Discussion:**

It is asserted that further comprehensive studies are required to support the hypothesis that ARB has the potential to improve oxidative and inflammatory lung injury via SIRT1/Nrf2 modulation.

## 1 Introduction

Methotrexate (MTX), a folate antagonist, is utilized in the treatment of a number of medical conditions, including leukemia, lung, breast and uterine cancers, as well as ectopic pregnancy and rheumatoid arthritis (1). The classical anticancer mechanism of action for MTX is to deplete cellular tetrahydrofolate pools by inhibiting dihydrofolate reductase (DHFR), thereby stopping DNA synthesis and thus cell proliferation in cancer cells (2). In addition, reduction of NADH/NADPH production via MTX antagonism of NADPH-dependent oxidoreductases and subsequent glutathione (GSH) depletion has been proposed as a second mechanism of its cytotoxicity (3). Despite its recognized effectiveness in treating numerous cancers, MTX can induce adverse effects, notably in healthy tissues exhibiting high metabolic activity (1, 4). It is generally administered in higher doses in the context of cancer chemotherapy, a practice that can result in toxicity and the onset of side effects, including bone marrow suppression, pulmono-, nephro- and hematological toxicities (1). It has been documented that treatment with MTX carries an elevated risk of developing lung disease, with MTX-related pulmonary toxicity potentially manifesting in the form of fibrosis, interstitial pneumonitis, and alveolar damage (4). Despite the absence of a comprehensive understanding of the mechanism of MTX toxicity, molecular studies have demonstrated that oxidative stress (OS) and inflammatory reactions are pivotal factors (5). It is established that MTX induces oxidative tissue damage of a severe nature by inhibiting antioxidant enzymes, including superoxide dismutase (SOD) and glutathione peroxidase (GPx), depleting the GSH pool, and inducing lipid peroxidation (LPO) (3, 6). The inflammatory response to MTX is initiated by pro-inflammatory cytokines, including interleukin-6 (IL-6) and tumor necrosis factor-alpha (TNF-α), which are known to increase dramatically as a consequence of the activation of the nuclear factor-kappa B (NF-κB) pathway (5). Chronic inflammation due to increased reactive oxygen species (ROS) and NF-κB activation eventually disrupts endoplasmic reticulum (ER) homeostasis and ER stress (ERS) occurs. All these processes act as triggers for apoptotic cell death and cause permanent lung damage (6, 7). Sirtuin 1 (SIRT1) is a NAD^+^-dependent deacetylase and functions as a transcription factor in various cellular processes, such as metabolism, OS, inflammation, apoptosis and cell cycle (8). It is known that inhibition of SIRT1 aggravates the degree of OS and inflammation (9). In addition to SIRT1, the nuclear factor erythroid-related factor 2 (Nrf2) pathway has been shown to play a crucial role in the elimination of ROS and inflammation-induced cellular damage (8). The extant body of experimental evidence has revealed a direct link between SIRT1 and Nrf2 signaling, thus demonstrating that both proteins exert a regulatory influence on each other’s activity (9). Over the past few years, significant progress has been made in the understanding of the molecular mechanisms underlying MTX-induced tissue damage, with a particular focus on the role of suppressed SIRT1/Nrf2 signaling (10, 11). This has led to a growing focus on identifying molecules capable of counteracting this inhibition, which is of strategic importance in the development of effective therapeutic interventions to mitigate MTX-induced tissue damage (7, 10).

Arbutin (ARB) is a hydroquinone glycoside that has been detected in a variety of plants, including members of the Asteraceae, Ericaceae, and Rosaceae families (12). It has demonstrated a number of biological activities, including antimicrobial, antihyperlipidemic, antioxidant, and anti-tumor properties (13). Experimental findings also indicate the capacity of ARB to influence the SIRT1 and Nrf2 pathways (14, 15). The aforementioned beneficial biological properties of ARB have resulted in an increase in its industrial application on an annual basis (12, 13). Experimental data have also been found suggesting a protective role for ARB in lipopolysaccharide (LPS) (14) and *Mycoplasma gallinarum*-induced (16) lung injury models. Nonetheless, the impact of ARB on MTX-induced pulmonary toxicity remains ambiguous. The present study aimed to investigate whether ARB protects against MTX-induced lung injury biochemically and histologically, including the SIRT1/Nrf2 signaling.

## 2 Materials and methods

### 2.1 Drugs and chemicals

The MTX, ARB, carboxymethylcellulose (CMC), dimethyl sulfoxide (DMSO) phosphate-buffered saline (PBS) tablet, thiobarbituric acid (TBA), sulfuric acid, paraformaldehyde, ethanol, xylene, hematoxylin and eosin (H&E) solution were purchased from Sigma-Aldrich (St. Louis, MO, United States). MTX was dissolved in physiological serum and administered intraperitoneally, while ARB was dissolved in 0.5% CMC containing 5% DMSO and administered orally to the animals. The total oxidant status (TOS) kit was purchased from Rel Assay Diagnostics (Gaziantep, Türkiye). All ELISA kits utilized for biochemical measurements were procured from Fine Biotech Co., Ltd. (Wuhan, China), and measurements were conducted in strict accordance with the manufacturer’s guidelines.

### 2.2 Animals and treatments

Thirty healthy female Sprague–Dawley rats, with a weight range of 170–180 g and aged between 8 and 10 weeks, were procured from the Surgical Practice Research Center of Karadeniz Technical University. The rats were housed within the same facility, under standard laboratory conditions that were maintained at a temperature between 22 and 24 °C and a humidity level between 40 and 70%, with free access to food and water. Following a 7-day acclimation period, the rats were randomly assigned to one of five groups of six rats each: control, high-dose ARB, MTX, MTX + low-dose ARB and MTX + high-dose ARB. The rats in the control group were administered 0.5% CMC orally for a period of 7 days and received an intraperitoneal saline injection on the second day. The rats in the high-dose ARB group were administered high dose ARB (100 mg/kg) orally for a period of 7 days and received an intraperitoneal saline injection on the second day. The rats in the MTX group were administered 0.5% CMC orally for a period of 7 days and received an intraperitoneal MTX (20 mg/kg) injection on the second day. The administered MTX dose was determined through consideration of the dose employed in previous experimental MTX-induced lung toxicity models (7, 17). The rats in the MTX + low-dose ARB group were administered ARB (50 mg/kg) orally for a period of 7 days and received an intraperitoneal MTX (20 mg/kg) injection on the second day. The rats in the MTX + high-dose ARB group were administered ARB (100 mg/kg) orally for a period of 7 days and received an intraperitoneal MTX (20 mg/kg) injection on the second day. The preferred ARB doses for this study were determined on the basis of prior research demonstrating the capacity of ARB to demonstrate antioxidant and anti-inflammatory properties in experimental models of ischemia/reperfusion-induced testicular injury (18) and complete Freund’s adjuvant-induced arthritis (19). On the morning of the 8th day of the experiment, all subjects were euthanized following overdose of ketamine and xylazine (4:1). Thereafter, the lung tissues of the subjects were retrieved for further analysis. Approval for this experimental study was granted by the Animal Research Ethics Committee of Karadeniz Technical University (Approval no: 2024/38), and the study was conducted in strict accordance with the ARRIVE guidelines.

### 2.3 Histological examination

Tissue samples were obtained from the same lobes of the rat lung tissues and fixed in 10% formaldehyde solution. Following dehydration via a graded series of alcoholic solutions, the samples were cleared with xylene and embedded in paraffin blocks. Following the staining of 5 μm sections from the paraffin blocks with H&E, they were subjected to evaluation and photography using a light microscope (Olympus BX51, Tokyo, Japan). The researcher conducting this analysis was unaware of the procedures applied to the groups (17). Furthermore, the presence of vascular congestion, hemorrhage, edema, leukocyte infiltration and apoptotic cell with hyperchromatic and pyknotic nuclei in five distinct regions, scanned at 200× magnification in a clockwise direction, was evaluated on a scale ranging from 0 to 4, with 0 representing a absence of such features and 4 indicating a widespread presence (20).

### 2.4 Tissue preparation

Following a rigorous washing process with ice-cold PBS to remove any contaminants, the lung tissues were then homogenized in PBS buffer (10% w/v; pH: 7.4) using an appropriate homogenizer. Following the obtaining of homogenates, the centrifugation process was initiated at 1,800 g, at a temperature of 4 °C, for a duration of 15 min. The protein content present within the collected supernatants was subsequently determined by means of the bicinchoninic acid method (21). Subsequently, the supernatants were utilized for biochemical analysis.

#### 2.4.1 Evaluation of the levels of LPO and TOS in lung tissue

The level of LPO in rat lung tissue was determined by means of a manual colorimetric method (22). In summary, 1 mL of supernatant was combined with TBA and sulfuric acid, and then heated at 100 °C for 60 min. Following this, the samples were cooled and then centrifuged at 1,800 g for 10 min. The degree of absorbance was measured at a wavelength of 532 nm, using a spectrophotometer (Molecular Devices, CA, United States) and the LPO levels in tissue were expressed with nmol malondialdehyde (MDA)/mg protein (23). The TOS levels in lung tissues were detected using a colorimetric kit in accordance with the kit instruction.

#### 2.4.2 Evaluation of the levels of antioxidant biomarkers in lung tissue

The GSH level in rat lung tissue was determined by means of the Ellman’s reagent-based a manual colorimetric kit (24). In summary, following the amalgamation of 100 μL of the supernatant and 100 μL of the Ellman’s reagent, the mixture was subjected to an incubation period at ambient temperature for a duration of 5 min. The subsequent step involved the measurement of the sample’s optical density at a wavelength of 412 nm using a spectrophotometer. The concentrations of GSH present within the tissue samples were expressed in terms of μmol/mg protein.

The levels of SOD, GPx, SIRT1, Nrf2 and heme oxygenase-1 (HO-1) in lung tissues were detected using rat-specific ELISA kits in accordance with the kit instructions. A conventional ELISA measurement comprises the following steps: Firstly, 100 μL of sample and standard solutions are transferred to a 96-well plate that has been coated with primary antibody. The plate is then incubated at 37 °C for 90 min. Subsequent to the completion of this period, the plate content is removed and washed. Then, 100 μL of biotin-labeled antibody solution is added to each well, and the plate is incubated at 37 °C for a further 60 min. Subsequent to the completion of this step, the plate content is removed and washed. Then, 100 μL of horseradish peroxidase solution is added to each well and the plate is incubated at 37 °C for 30 min. Subsequently, the plate content is removed and washed, then 90 μL of TMB substrate solution is added to each well and the plate is incubated at 37 °C for 20 min. Subsequent to the conclusion of this period, 50 μL of stop solution is added to each well, and the absorbances of the wells are measured at 450 nm using a spectrophotometer. A standard curve is plotted with absorbance values against standard concentrations. The amount of analyte in the samples is calculated by substituting the sample absorbances on the plotted graph of absorbances against standard concentrations (25).

#### 2.4.3 Evaluation of the levels of inflammatory biomarkers in lung tissue

The levels of NF-κB p65, IL-6 and myeloperoxidase (MPO) in lung tissues were detected using rat-specific ELISA kits in accordance with the kit instructions.

#### 2.4.4 Evaluation of the levels of ERS and apoptosis biomarkers in lung tissue

The levels of heat shock protein family A member 5 (HSPA5), activating transcription factor 6 (ATF6), growth arrest and DNA damage-inducible gene 153 (GADD153) and cleaved caspase-3 (CASP3) in lung tissues were detected using rat-specific ELISA kits in accordance with the kit instructions.

### 2.5 Statistical analysis

Data analysis was performed using the SPSS 23.0 program (Chicago, IL). Distribution of variables was evaluated using the Shapiro–Wilk test. ANOVA and *post-hoc* Tukey test were used in the statistical analysis of parametrically distributed variables. The results were expressed as mean±SEM. Differences at *p* < 0.05 were considered statistically significant.

## 3 Results

### 3.1 Effect of ARB on pulmonary OS and antioxidant parameters of the MTX-intoxicated rats

**Figure 1:**
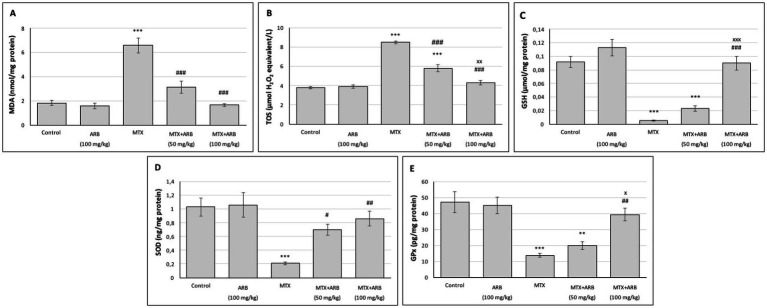
Effects of ARB on OS and antioxidant biomarkers in lung tissues of MTX-intoxicated rats. The levels of MDA **(A)**, TOS **(B)**, GSH **(C)**, SOD **(D)**, and GPx **(E)** in lung tissues. Values are expressed as mean ± SEM (*n* = 6). Compared with control group ^**^p < 0.01 and ^***^p < 0.001. Compared with MTX group ^#^*p* < 0.05, ^##^*p* < 0.01, and ^###^*p* < 0.001. Compared with MTX + ARB (50 mg/kg) group ^x^*p* < 0.05 and ^xxx^*p* < 0.001.

The data pertaining to OS and antioxidant biomarkers in the lung is illustrated in Figure 1. No statistically significant difference was observed between the control group and the ARB group. However, in comparison with the control group, the MTX group exhibited significantly elevated MDA and TOS levels, alongside significantly diminished GSH, GPx, and SOD levels. Conversely, ARB dose-dependently reduced OS and restored antioxidant defenses.

### 3.2 Effect of ARB on pulmonary SIRT1/Nrf2/HO-1 axis of the MTX-intoxicated rats

**Figure 2:**
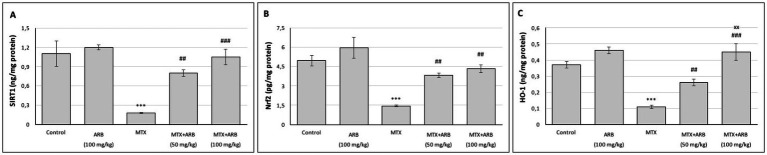
Effects of ARB on SIRT1 **(A)**, Nrf2 **(B)** and HO-1 **(C)** in lung tissues of MTX-intoxicated rats. Values are expressed as mean ± SEM (*n* = 6). Compared with control group ^***^p < 0.001. Compared with MTX group ^##^*p* < 0.01 and ^###^*p* < 0.001. Compared with MTX + ARB (50 mg/kg) group ^xx^*p* < 0.01.

The levels of SIRT1, Nrf2 and HO-1 in the lungs are illustrated in Figure 2. There was no significant difference between the control group and the rats of ARB group. However, when compared with the control group, the MTX group exhibited a significant suppression of lung SIRT1, Nrf2 and HO-1 levels. Conversely, ARB treatments significantly enhanced the levels of these proteins, with the 100 mg/kg ARB treatment group demonstrating greater efficacy compared to the 50 mg/kg ARB treatment group.

### 3.3 Effect of ARB on pulmonary inflammatory parameters of the MTX-intoxicated rats

**Figure 3:**
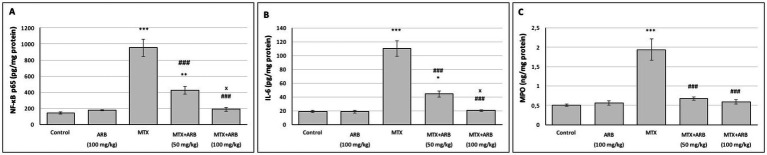
Effects of ARB on inflammatory biomarkers in lung tissues of MTX-intoxicated rats. The levels of NF-κB p65 **(A)**, IL-6 **(B)**, and MPO **(C)** in lung tissues. Values are expressed as mean ± SEM (*n* = 6). Compared with control group ^*^p < 0.05, ^**^p < 0.01, and ^***^p < 0.001. Compared with MTX group ^###^*p* < 0.001. Compared with MTX + ARB (50 mg/kg) group ^x^*p* < 0.05.

The levels of inflammatory biomarkers in lung tissues are presented in Figure 3. The results indicate that there is no statistically significant difference between the control group and the rats of the ARB group. However, when compared with the control group, administration of MTX resulted in a marked induction of lung inflammation. However, the administration of ARB in conjunction with MTX led to a substantial suppression of the level of lung inflammation.

### 3.4 Effect of ARB on pulmonary ERS and apoptosis parameters of the MTX-intoxicated rats

**Figure 4:**
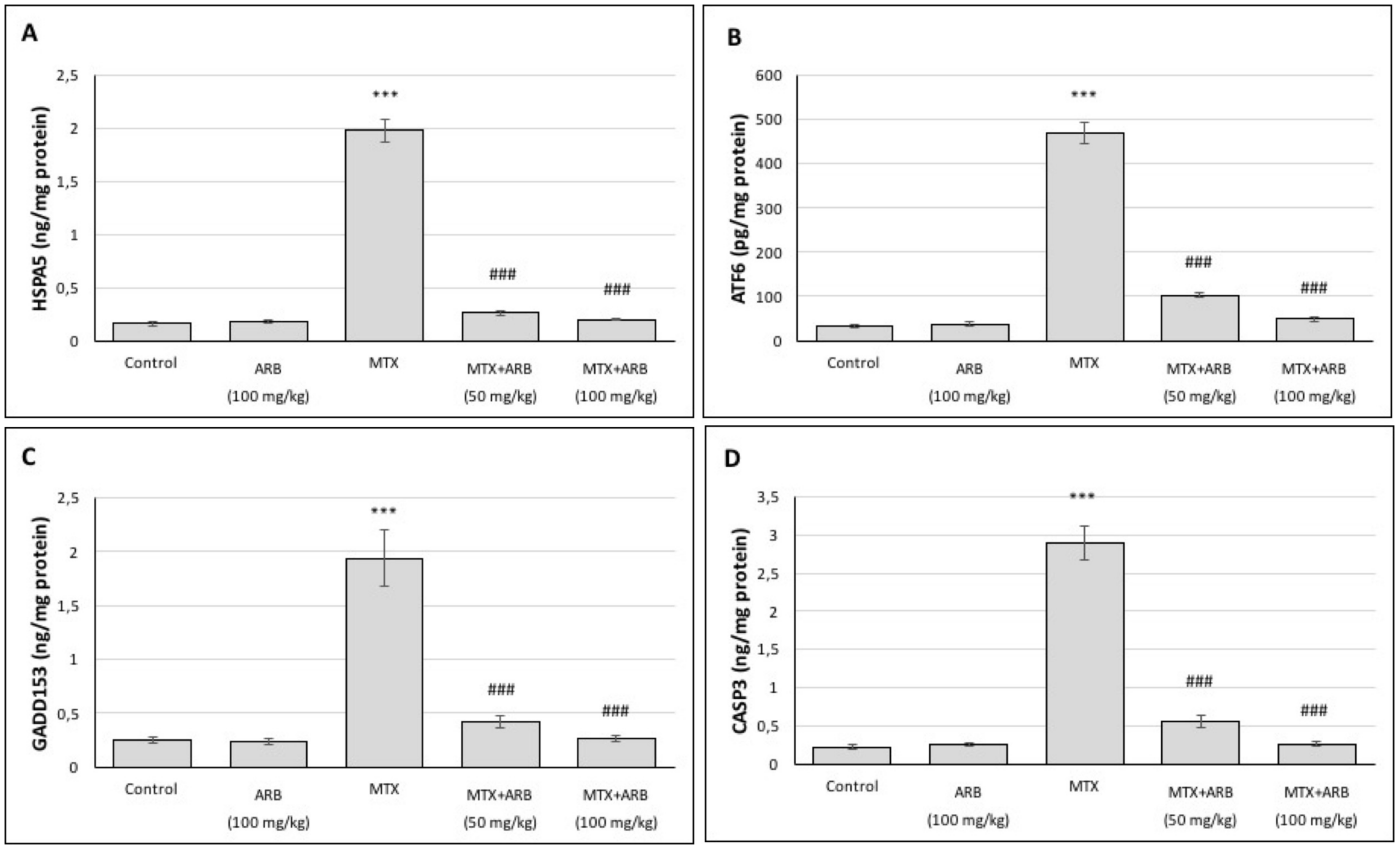
Effects of ARB on ERS and apoptosis biomarkers in lung tissues of MTX-intoxicated rats. The levels of HSPA5 **(A)**, ATF6 **(B)**, GADD153 **(C)**, and CASP3 **(D)** in lung tissues. Values are expressed as mean ± SEM (*n* = 6). Compared with control group ^***^p < 0.001. Compared with MTX group ^###^*p* < 0.001.

The levels of ERS and apoptosis in the lung tissues are presented in Figure 4. The results indicate that there is no statistically significant difference between the control group and the rats of the ARB group. However, administration of MTX significantly induced ERS and apoptosis levels in comparison with the control group. Conversely, the combination of ARB treatment with MTX administration led to a substantial improvement in lung ERS and apoptosis levels.

### 3.5 Effect of ARB on pulmonary histopathological findings of the MTX-intoxicated rats

**Figure 5:**
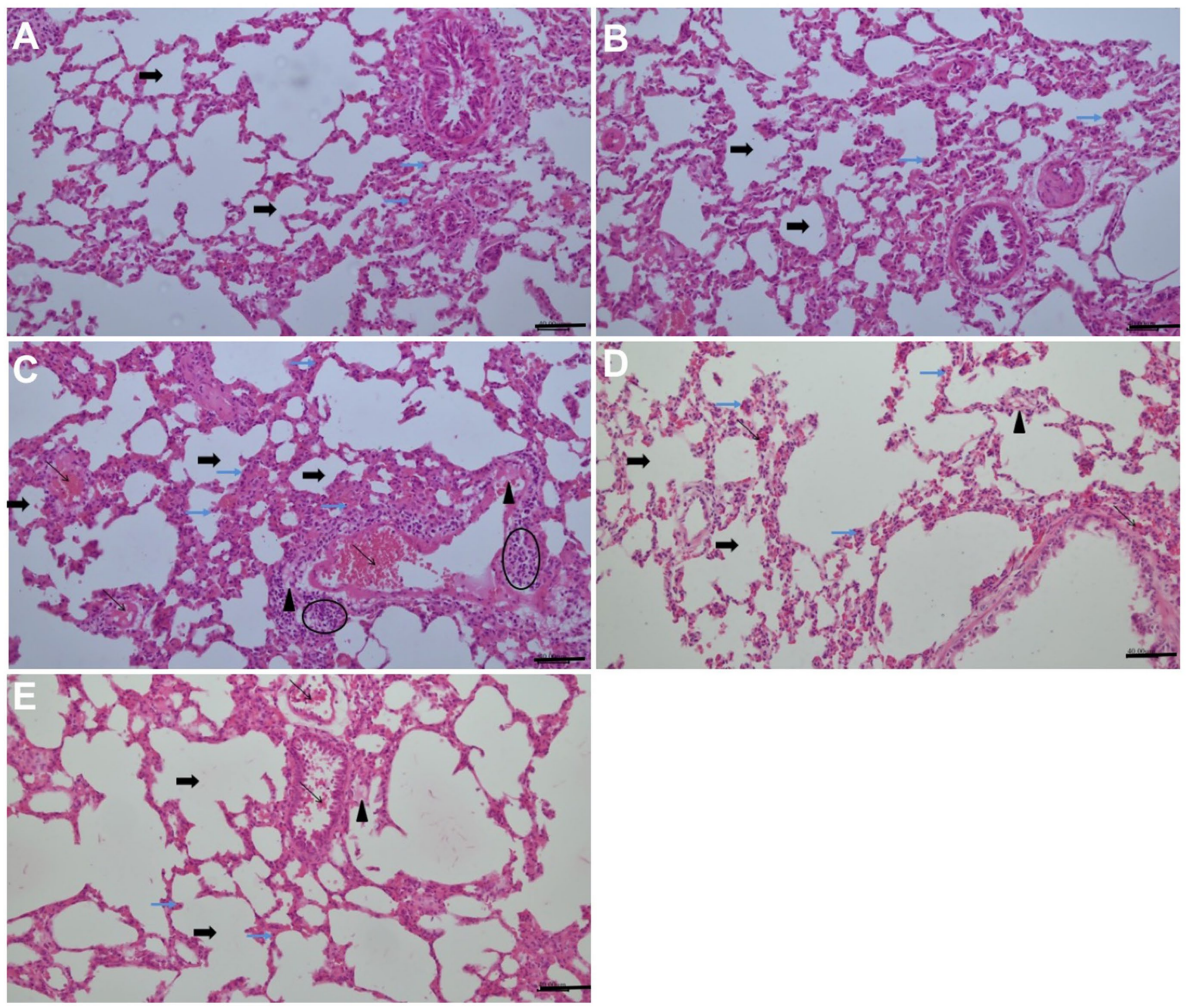
Effects of ARB treatment on the lung architecture in MTX-intoxicated rats (H&E staining, 200×, Scale bars = 40 μm). **(A)** The lung sections of the control group exhibited regular morphology in terms of both alveolar wall thickness and general lung structure, and apoptotic cells with hyperchromatic and pyknotic nuclei were present at the basal level. **(B)** In the only high-dose ARB group, lung sections with a regular morphology that closely resembled normal structures. There was mild alveolar thickening and vascular congestion in specific locations, and apoptotic cells with hyperchromatic and pyknotic nuclei were present at the basal level. **(C)** Widespread alveolar wall thickening, moderate vascular congestion, inflammatory cell infiltration, oedema and elevated apoptotic cells with hyperchromatic and pyknotic nuclei were obtained in the lung sections of the MTX group. **(D)** Lung sections from the MTX + ARB (50 mg/kg) group showed signs of oedema in the interalveolar area and mild vascular congestion between the alveoli. The alveolar structure displayed characteristics that almost resembled normal appearance. Although fewer than in the MTX group, apoptotic cells with hyperchromatic and pyknotic nuclei were also present. **(E)** Lung sections from the MTX + ARB (100 mg/kg) group exhibited characteristics that closely resembled normal lung tissue, with localized oedema and alveolar thickening in the interalveolar area. There were also a small number apoptotic cells with hyperchromatic and pyknotic nuclei, similar to those observed in the control group. Thick black arrow: alveol, thin black arrow: vascular congestion, thin blue arrow: apoptotic cells with hyperchromatic and pyknotic nuclei, arrowhead: edema, elliptic: inflammatory cell infiltration.

In the lung sections of the control group, the thickness of the alveolar wall and the general structure of the lungs exhibited regular morphology. In the only high-dose ARB group, the lung sections exhibited a regular morphology, closely resembling normal structures, with mild alveolar thickening and vascular congestion in specific locations. In the lung sections of the MTX group, widespread alveolar wall thickening, moderate vascular congestion, inflammatory cell infiltration and edema findings were obtained. The lung sections from the MTX + ARB (50 mg/kg) group exhibited signs of edema in the interalveolar area and mild vascular congestion between the alveoli, with the alveolar structure displaying characteristics that nearly resembled a normal appearance. The lung sections from the MTX + ARB (100 mg/kg) group exhibited characteristics that nearly resembled a normal appearance, with edema and alveolar thickening in places in the interalveolar area (Figure 5).

MTX administration resulted in a marked increase in semi-quantitative vascular congestion, hemorrhage and injury scores in comparison with the control group, while ARB administration led to a significant improvement in these scores (Table 1).

**Table 1:** Effect of ARB on histopathological changes in the lung tissue of MTX-intoxicated rats.

	Control	ARB (100 mg/kg)	MTX	MTX + ARB (50 mg/kg)	MTX + ARB (100 mg/kg)
Vascular congestion	0.33 ± 0.21	0.50 ± 0.22	2.50 ± 0.22^***^	1.17 ± 0.17^##^	0.67 ± 0.21^###^
Hemorrhage	0.33 ± 0.21	0.50 ± 0.22	1.50 ± 0.22^**^	0.66 ± 0.21	0.50 ± 0.22^#^
Injury score	0.33 ± 0.21	0.67 ± 0.21	2.50 ± 0.22^***^	0.83 ± 0.17^###^	0.67 ± 0.33^###^

ARB, arbutin; MTX, methotrexate. Data were expressed as mean ± SEM (*n* = 6). *p*-values according to ANOVA and *post-hoc* Tukey test. Compared with control group: ^**^p < 0.01 and ^***^p < 0.001. Compared with MTX group: ^#^*p* < 0.05, ^##^*p* < 0.01, and ^###^*p* < 0.001.

## 4 Discussion

The MTX is utilized in the management of inflammatory diseases and various malignancies; however, its propensity to induce lung toxicity represents a significant constraint on its therapeutic application (1, 4). The present study concentrated on appraising the protective efficacy of ARB (a renowned antioxidant and anti-inflammatory agent) in counteracting the pulmonary toxicity of MTX by biochemical and histological methods. The utilization of experimental animals is a prevalent practice, particularly within the domain of investigating the mechanism of organ toxicity associated with MTX. In the course of such investigations, the establishment of a toxicity model generally entails the administration of a single intraperitoneal dose of MTX at an initial concentration of 20 mg per kg of body mass (7, 17). Consequently, in the present study, intraperitoneal MTX administration was performed on day 2 in the MTX groups. In accordance with the findings of earlier research (7, 26, 27), the results demonstrated that rats administered with MTX exhibited histopathological findings indicative of oxidative and inflammatory damage in their lung tissues. This finding was interpreted as an indication that the MTX-induced pulmonotoxicity model had been successfully established. However, the administration of ARB treatments in combination with MTX (particularly at a dose of 100 mg/kg) resulted in a significant improvement in the histopathological findings in lung tissue. These results were consistent with the findings of previous studies which demonstrated that ARB was capable of exerting a lung-protective effect through the reduction of inflammatory cell infiltration in a model of lung injury induced by LPS (14), as well as through the improvement of inflammatory cell infiltration, congestion, and bleeding findings in a model of lung injury induced by *Mycoplasma gallinarum* (16).

The OS has been identified as a primary initiating mechanism in tissue damage caused by pathological stimuli. Over time, this stress can induce inflammation, ERS and cell death pathways, which can ultimately lead to organ failure (6). It is widely accepted that increased OS is the primary cause of MTX-induced pulmonary toxicity (5). A body of experimental studies conducted to date has indicated that the administration of MTX leads to an augmentation in the production of ROS, with a particular emphasis on the formation of superoxide radical (1, 3). It has been demonstrated that excessive ROS instigates LPO and results in the generation of toxic and mutagenic by-products, including MDA, while concurrently inducing structural damage to the cell’s antioxidant proteins SOD and GPx. Consequently, the presence of these mechanisms results in the occurrence of oxidative damage induced by MTX (5). The most significant regulatory factor in the process of cellular responses to stress factors is Nrf2 (28). In the event of cellular toxicity, increased ROS abolish the Nrf2/Kelch-like ECH-associated protein 1 interaction, and the nuclear translocation of released Nrf2 induces the expression of numerous antioxidant genes, including SOD, HO-1, and GPx (28). SIRT1, a member of the histone deacetylase family, plays a pivotal role in the regulation of OS and mitochondrial metabolism (29). In line with these informations, our research demonstrated that administration of MTX led to an increase in LPO levels in lung tissue, which was a result of the inhibition of the SIRT1/Nrf2 axis and the suppression of the antioxidant system. These results were in accordance with those of previous research, which has shown that MTX causes oxidative damage to tissue by inhibiting the SIRT1 and Nrf2 pathways (5, 10, 11). However, the application of ARB in conjunction with MTX resulted in a substantial suppression of LPO and TOS levels, achieved through the regeneration of the antioxidant system by modulating SIRT1 and Nrf2 in a dose-dependent manner. The radical scavenging activity of ARB is well documented (13). In this study, the reduction in MTX-induced OS levels in the ARB treatment groups is hypothesized to be due to the direct radical scavenging activity of ARB and a synergistic effect of the previously demonstrated SIRT1/Nrf2 modulatory effects of ARB (14, 25, 30).

Inflammation is defined as a physiological response of the body to pathological stimuli, which activates three mechanisms: namely, an increased perfusion of the affected tissue, an increased permeability of the capillaries, and an elevated leukocyte infiltration (indicating higher MPO levels) (5). A plethora of preceding investigations have demonstrated that inflammation, characterized by elevated levels of pro-inflammatory cytokines, is a contributing factor to MTX-induced tissue injury (10, 11). The NF-κB is a pivotal regulator that plays a critical role in the activation of numerous inflammatory cytokines, such as IL-6 and TNF-α (31). In this study, MTX treatment activated the NF-κB cascade in lung tissue and induced a strong inflammatory response, in agreement with previous studies (5, 32). Interestingly, the inflammatory response induced by MTX in lung tissue was greatly attenuated by the administration of ARB. The identification of a negative regulation between Nrf2 and NF-κB pathways confirmed the hypothesis that a reduction in ROS production could lead to a reduction in inflammation (5). Additionally, SIRT1 has been shown to suppress the inflammatory response by hindering NF-κB signaling (8). In light of this information, it is suggested that the mechanism by which ARB treatment reduces MTX-induced lung damage may involve the anti-inflammatory activity of ARB resulting from their antioxidant and SIRT1/Nrf2 modulator properties. This hypothesis is supported by the lower levels of OS and higher levels of SIRT1/Nrf2 in the ARB groups compared with the MTX group. In addition, this situation is in line with the results of the anti-inflammatory activity of the ARB previously demonstrated in experimental models of LPS-induced lung injury (14) and in cyclophosphamide-induced liver injury (30).

The ER is a multi-functional organelle within the cell that is responsible for the synthesis, folding and quality control of proteins (33). Insults like OS, toxins, ischemia and inflammation impair the folding capacity of ER proteins in cells, and the unfolded proteins build up in the ER lumen, leading to ERS (34). Cells activate the unfolded protein response (UPR), which activates mechanisms, such as inhibition of protein synthesis, regulation of gene expression and apoptosis, in order to restore proteostasis by eliminating the ERS (35). HSPA5, ATF6 and GADD153 are ERS biomarker proteins frequently used to determine ERS level (7). Recent reports have highlighted the role of increased ERS and ERS-induced apoptosis in MTX-associated tissue damage (7, 36). In line with these reports, the results of our study showed that the administration of MTX induced the levels of the biomarkers of ERS and apoptosis in the lung tissue. A growing body of evidence points to the possibility of cross-regulation of ERS components and SIRT1 expression (34). Activation of SIRT1 reduces the level of OS and inflammation, which also alleviates the ERS (33). Nrf2 is also known to regulate the expression of proteins responsible for removing unfolded proteins by proteasomal degradation (35). These results are supported by our findings of increased ERS and apoptosis in MTX despite decreased SIRT1/Nrf2. However, ERS and apoptosis levels were significantly abolished by ARB treatment in combination with MTX. These results were in line with previous studies showing that ARB could suppress ERS and apoptosis in models of LPS-induced renal injury (36) and ischaemia/reperfusion-induced testis injury (18).

## 5 Conclusion

The results of this study demonstrate for the first time that ARB can play a protective role in an experimental model of MTX-related pulmonary toxicity, reducing OS, inflammatory and ERS mechanisms. This protective effect of ARB was found to be mediated at least in part through the regulation of the SIRT1/Nrf2 pathway. ARB shows potential as an adjuvant to reduce MTX-induced pulmonary toxicity, warranting further preclinical and clinical evaluation.

## Data Availability

The raw data supporting the conclusions of this article will be made available by the authors, without undue reservation.

## Glossary

ARB: Arbutin
ATF6: Activating transcription factor 6
CASP3: Cleaved caspase-3
CMC: Carboxymethylcellulose
DHFR: Dihydrofolate reductase
DMSO: Dimethyl sulfoxide
ERS: Endoplasmic reticulum stress
GADD153: Growth arrest and DNA damage-inducible gene 153
GPx: Glutathione peroxidase
GSH: Glutathione
H&E: Hematoxylin and eosin
HO-1: Heme oxygenase-1
HSPA5: Heat shock protein family A member 5
IL-6: Interleukin-6
LPO: Lipid peroxidation
LPS: Lipopolysaccharide
MDA: Malondialdehyde
MTX: Methotrexate
MPO: Myeloperoxidase
NF-κB: Nuclear factor kappa B p65
Nrf2: Nuclear factor erythroid 2-related factor 2
OS: Oxidative stress
PBS: Phosphate-buffered saline
ROS: Reactive oxygen species
SEM: Standard error of the mean
SIRT1: Sirtuin 1
SOD: Superoxide dismutase
TBA: Thiobarbituric acid
TNF-α: Tumor necrosis factor-alpha
UPR: Unfolded protein response
